# Assessing HER2 amplification in breast cancer: findings from the Australian In Situ Hybridization Program

**DOI:** 10.1007/s10549-012-2093-6

**Published:** 2012-06-08

**Authors:** Michael Bilous, Adrienne L. Morey, Jane E. Armes, Richard Bell, Peter H. Button, Margaret C. Cummings, Stephen B. Fox, Glenn D. Francis, Brigid Waite, Glenda McCue, Wendy A. Raymond, Peter D. Robbins, Gelareh Farshid

**Affiliations:** 1 Healthscope Pathology, The Norwest Private Hospital, Bella Vista, Sydney, NSW 2153 Australia; 2 ICPMR Westmead Hospital and The University of Sydney, Sydney, NSW 2145 Australia; 3 Sydpath, St Vincent’s Hospital, Sydney, NSW 2010 Australia; 4 Anatomical Pathology, Mater Health Services, South Brisbane, QLD 4011 Australia; 5 The Andrew Love Cancer Centre, 70 Swanston Street, Geelong, VIC 3250 Australia; 6 Infopeople Pty Limited, Mezzanine Level, 383 Kent Street, Sydney, NSW 2000 Australia; 7 University of Queensland Centre for Clinical Research and Pathology Queensland, Royal Brisbane and Women’s Hospital, Herston, QLD 4029 Australia; 8 The Peter MacCallum Cancer Centre and the University of Melbourne, St Andrews Place, East Melbourne, VIC 3002 Australia; 9 Pathology Queensland, Herston Hospitals Campus, Royal Brisbane and Women’s Hospital, Herston Rd, Herston, QLD 4029 Australia; 10 Molecular and Clinical Pathology Research Laboratory, Princess Alexandra Hospital, Ipswich Rd, Woolloongabba, QLD 4102 Australia; 11 Griffith University, Gold Coast, QLD Australia; 18 University of Queensland, St Lucia, QLD 4222 Australia; 12 Roche Products Pty Limited, 4–10 Inman Road, Dee Why, NSW 2099 Australia; 13 Woolomin Consulting, “Woolomin”, 2535 Sapphire Road, Wheeo, NSW 2583 Australia; 14 Healthscope Pathology, 1 Goodwood Rd, Wayville, SA 5034 Australia; 15 Flinders Medical Centre, Bedford Park, Adelaide, SA 5042 Australia; 16 Tissue Pathology, PathWest QEII Medical Centre, Locked Bag 2009, Nedlands, WA 6008 Australia; 17 Divisions of Surgical Pathology & Cytopathology, SA Pathology, Adelaide, SA 5006 Australia

**Keywords:** Breast cancer, HER2 Genes, In situ hybridization, Immunohistochemistry

## Abstract

In August 2006, the Australian government approved subsidized trastuzumab therapy for human epidermal growth factor receptor 2 (HER2)-positive early breast cancer, and it was mandated that HER2 testing should be performed using in situ hybridization (ISH) rather than immunohistochemistry (IHC). Here we review results of the first regulated, nationwide program to provide HER2 ISH testing for all newly diagnosed breast cancer patients, with a particular emphasis on cases where IHC and ISH results were discordant. Data from all laboratories participating in the program were collated. Cases with an equivocal ISH test result [by chromogenic ISH (CISH) or silver ISH (SISH)] were tested centrally by fluorescence ISH. Most laboratories also performed HER2 IHC, and 200 cases with discordant IHC and ISH results were selected for further analysis in a central laboratory. A total of 26 laboratories were involved and 53,402 tests were reported. Over a 4-year period the HER2 positivity rate decreased for primary cancers from 23.8 to 14.6 %, but remained relatively constant for samples from metastases. Average ISH reporting times were <5 days for all yearly reporting periods. Test-repeat rates decreased for CISH (8.9–3.6 %) and SISH (13.7–8.4 %). Only 12 of 196 cases remained discordant after retesting in a central laboratory. These findings demonstrate the successful implementation of a regulated, national program that continues to collect data on HER2 status. The results also highlight the differences in IHC interpretation between local laboratories and a central, more experienced, laboratory. This model could be used to establish future biomarker-testing programs in other countries.

## Introduction

The human epidermal growth factor receptor 2 (*HER2*) gene is amplified in ~15–20 % of breast cancers and has been linked with poor prognosis [1–6], making it an attractive molecular target for breast cancer therapy. Trastuzumab (Herceptin^®^; F. Hoffmann-La Roche Ltd, Basel, Switzerland) is an anti-HER2 monoclonal antibody with proven survival benefits in the treatment of women with HER2-positive metastatic breast cancer (MBC) [7–11] and early breast cancer (EBC) [12–15]. Evaluation of the HER2 status of all breast cancers at diagnosis is recommended to predict the potential benefit from trastuzumab treatment [16, 17].

HER2 testing is performed by either immunohistochemistry (IHC) or in situ hybridization (ISH). IHC uses anti-HER2 antibodies to detect HER2 protein expression levels, and is assessed semiquantitatively by the proportion and intensity of staining. ISH uses DNA probes to determine *HER2* gene copy number. To ensure accurate HER2 testing, as well as consistent and appropriate patient selection for trastuzumab therapy, the American Society of Clinical Oncology (ASCO) and the College of American Pathologists (CAP) convened an expert panel to compile and publish HER2 testing recommendations that included an algorithm to define positive, negative, and equivocal HER2 results according to both HER2 protein expression and gene amplification [18]. According to the ASCO/CAP guidelines, a HER2-positive result by IHC is uniform, intense staining of >30 % of invasive tumor cells (3+) and a positive result by ISH is >6 *HER2* gene copies per nucleus or a *HER2* gene:chromosome enumeration probe 17 (CEP17) signal ratio of >2.2 [18].

A minority of the ASCO/CAP panel expressed the view that IHC is not a sufficiently accurate assay to determine HER2 status [18], and two large trials have shown discordance between local and central HER2 testing by IHC [19] or by both IHC and fluorescence ISH (FISH) [20]. Analysis of concordance between a local and a high-volume central laboratory in a phase IV trial [21] also showed poor concordance of IHC results, and concluded that HER2 testing is most accurate when performed at a high-volume central laboratory.

In Australia, ~14,000 new breast cancer cases are diagnosed annually [22]. Patients with HER2-positive MBC, determined by either IHC or ISH, are eligible for trastuzumab therapy as part of the Herceptin program administered by Medicare Australia. Patients with HER2-positive EBC are also eligible for trastuzumab therapy under the Australian government-funded Pharmaceutical Benefits Scheme. The Pharmaceutical Benefits Advisory Committee specified that HER2 positivity should be demonstrated by ISH in these patients. This requirement led to the development of the Australian In Situ Hybridization Program, a nationwide program utilizing ISH as the HER2 testing platform. The program was launched as a multicenter, coordinated project, with the primary objective being to provide accurate tumor ISH testing for all patients diagnosed with EBC. Accurate testing is critical in guiding the provision of trastuzumab therapy to those who are likely to derive the most benefit from the treatment.

Here, we include details of the HER2 positivity rates recorded across Australia from October 2006 to September 2010 for patients with EBC or MBC, along with other test-related data; including result turnaround times and repeat testing rates. Most laboratories continue to use IHC in parallel with ISH and we also document the results of a reevaluation of 200 samples that had shown discordance between local IHC results and ISH results from a central reference laboratory.

## Methods

### Study design

The Australian In Situ Hybridization Program is a nationwide, multicenter, coordinated project sponsored by Roche Products Pty Limited (Dee Why, Australia) and overseen by the Australian HER2 Testing Advisory Board. Details of establishing of this program, including identification, training, certification, and accreditation of all laboratories, as well as the implementation of standardized reporting protocols, have been described previously [22].

### Sample selection

The majority of samples for HER2 ISH testing were from excised tumors from women aged ≥18 years with EBC or MBC. Approximately 10 % of samples were core biopsies and <1 % were from fine needle aspiration cell block material, or from male breast cancer patients.

### HER2 testing

**Fig. 1:**
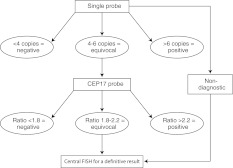
In situ hybridization assay algorithm for determining human epidermal growth factor receptor 2 status and eligibility for trastuzumab therapy in Australia. *CEP17* chromosome enumeration probe 17, *FISH* fluorescence in situ hybridization

All local laboratories were responsible for the provision of an accurate and timely HER2 testing service to support clinical decision-making in their area. Figure 1 shows the ISH assay algorithm used to determine HER2 positivity. A validated single-probe ISH test was used for all samples, with a CEP17 probe used for equivocal cases, defined as 4–6 *HER2* signals per nucleus. Cases that remained equivocal following dual-probe testing (defined as a *HER2*:CEP17 ratio of 1.8–2.2) or which were non-diagnostic due to a weak signal, were sent to a central reference laboratory for FISH testing using the PathVysion^®^ kit (Vysis/Abbott, Illinois, USA). IHC was used in conjunction with ISH as a quality control, both to assess tumor heterogeneity and to assist in the overall assessment of difficult-to-assess cases. In the initial phase of the program, IHC was used by some laboratories lacking the facility to perform ISH, to triage cases to be sent for ISH testing at one of the program laboratories. This practice gradually diminished over time, such that the vast majority of invasive cancer cases were submitted for ISH testing regardless of whether IHC had been performed, or of the IHC result.

All laboratories initially used a chromogenic ISH kit (CISH; SPoT-Light^®^ CISH, Invitrogen, California, USA). Approximately 1 year after the launch of the program, silver ISH (SISH; Inform^™^, Ventana Medical Systems, Inc., Arizona, USA) was also included as an alternative ISH testing assay, with a third option (DuoCISH^™^, Dako, Glostrup, Denmark) included 6 months later. All kits were used in accordance with the ISH assay algorithm (Fig. 1) and, from March 2008, the scoring of all HER2 tests adhered to the 2007 ASCO/CAP recommendations [18].

### Data collection

All HER2 test results, reporting times, test-repeat rates, and the proportion of tests performed on core biopsies were recorded. Means of each parameter were calculated for each laboratory and state during the measurement periods of October–September for 2006–2007, 2007–2008, 2008–2009, and 2009–2010. Mean HER2 positivity rates were also calculated for each laboratory and state for the four 12-month time periods.

### Comparison of IHC and ISH results

Two hundred invasive carcinomas were selected from patients, in which IHC had been performed at a local laboratory and the paraffin blocks or unstained sections had been forwarded to a central reference laboratory for ISH testing. All selected cases had shown discordance between local IHC and central ISH results. The cases included were either IHC 0/IHC 1+ that showed *HER2* gene amplification (false-negative, *n* = 31) or IHC 3+ that showed no *HER2* gene amplification (false-positive, *n* = 169). The central laboratory performed IHC on archived, unstained sections from all the cases using the 4B5 antibody (Ventana Medical Systems, Inc.) on the Ventana BenchMark Immunostainer (Ventana Medical Systems, Inc.). The stained slides were then assessed by a pathologist blinded to both the original IHC result, and to the *HER2* gene amplification status. In cases of uncertainty a second pathologist independently assessed the slide and, if necessary, the case was viewed by both pathologists together and a consensus score reached.

## Results

### HER2 positivity rates

By September 2010, the Australian In Situ Hybridization Program had been running for 4 years and had performed 53,402 ISH tests. Overall, 26 laboratories were approved for ISH testing. In the final reporting period, eight laboratories used CISH and 18 used SISH assays.

The total number of ISH tests conducted between October 2006 and September 2010, as well as the HER2 positivity rates for samples from EBC and MBC, are shown in Tables 1 and 2. Between October 2006 and September 2010, the overall HER2 ISH positivity rate was 16.9 % for EBC and 22.5 % for MBC. The HER2 positivity rate for EBC decreased each year from 23.8 % in the first 12-month period to 14.6 % in the final 12 months, whereas the MBC HER2 positivity rate varied from 22.6 % in the first 12-month period to 25.1, 21.3, and 21.6 % in the second, third, and fourth 12-month periods, respectively.

**Table 1:** HER2 positivity rates by ISH in early breast cancer tissue samples

State	October 2006 –September 2007		October 2007–September 2008		October 2008–September 2009		October 2009–September 2010	
	Total cases, *n*	ISH-positive, *n* (%)	Total cases, *n*	ISH-positive, *n* (%)	Total cases, *n*	ISH-positive, *n* (%)	Total cases, *n*	ISH-positive, *n* (%)
Australian Capital Territory	208	34 (16.3)	310	54 (17.4)	352	67 (19.0)	371	53 (14.3)
New South Wales	3,424	989 (28.9)	5,025	995 (19.8)	6,067	1,021 (16.8)	6,076	870 (14.3)
Queensland	1,423	356 (25.0)	2,205	330 (14.9)	2,811	410 (14.6)	3,151	468 (14.9)
South Australia	827	124 (15.0)	1,060	153 (14.4)	1,254	181 (14.4)	1,247	147 (11.8)
Victoria	1,478	306 (20.7)	2,391	360 (15.1)	2,751	464 (16.9)	3,879	660 (17.0)
Western Australia	475	56 (11.8)	704	86 (12.2)	1,455	181 (12.4)	1,571	176 (11.2)
Tasmania	–	–	88	11 (12.5)	312	48 (15.4)	292	51 (17.5)
Northern Territory^a^	–	–	–	–	–	–	–	–
Total	7,835	1,865 (23.8)	11,783	1,989 (16.8)	15,002	2,372 (15.8)	16,587	2,425 (14.6)

Results from states across Australia recorded over time

*HER2* human epidermal growth factor receptor 2, *ISH* in situ hybridization

^a^Northern Territory cases were tested at an ISH reference laboratory in New South Wales

**Table 2:** HER2 positivity rates by ISH in metastatic breast cancer tissue samples

State	October 2006–September 2007		October 2007–September 2008		October 2008–September 2009		October 2009–September 2010	
	Total cases, *n*	ISH-positive, *n* (%)	Total cases, *n*	ISH-positive, *n* (%)	Total cases, *n*	ISH-positive, *n* (%)	Total cases, *n*	ISH-positive, *n* (%)
Australian Capital Territory	–	–	3	0 (0.0)	12	2 (16.7)	4	0 (0.0)
New South Wales	513	116 (22.6)	336	92 (27.4)	279	63 (22.6)	226	48 (21.2)
Queensland	–	–	55	12 (21.8)	75	14 (18.7)	85	20 (23.5)
South Australia	–	–	14	5 (35.7)	61	13 (21.3)	96	14 (14.6)
Victoria	–	–	54	8 (14.8)	123	27 (22.0)	139	27 (19.4)
Western Australia	–	–	11	2 (18.2)	34	6 (17.7)	44	21 (47.7)
Tasmania	–	–	–	–	8	1 (12.5)	23	3 (13.0)
Northern Territory^a^	–	–	–	–	–	–	–	–
Total	513	116 (22.6)	473	119 (25.1)	592	126 (21.3)	617	133 (21.6)

Results from states across Australia recorded over time

*HER2* human epidermal growth factor receptor 2, *ISH* in situ hybridization

^a^Northern Territory cases were tested at an ISH reference laboratory in New South Wales

The majority of tumor specimens used for HER2 testing were obtained from excised tumors. The proportion of core biopsy samples tested remained consistently low and rarely exceeded 10 %. Testing of core biopsies was actively discouraged unless the HER2 status was required for a clinical decision regarding neoadjuvant therapy.

Reporting time data were provided by 17 of 18 laboratories in the first 12 months, 20 of 22 laboratories in the second 12 months, and all 26 laboratories in the final two 12-month periods. The average ISH reporting time from the date of the request for a HER2 test remained relatively unchanged between the reporting periods (4.9, 4.7, 4.6, and 4.5 days, respectively). For individual laboratories, average reporting times ranged from 1.3 to 12.9 days in the first 12 months, from 1.6 to 10.5 days in the second 12 months, from 1.0 to 10.2 days in the third 12 months, and from 1.3 to 10.9 days in the final 12 months. Average reporting times were longer than 7 days for 2 out of 17, 4 out of 20, 6 out of 26, and 4 out of 26 laboratories for the four consecutive reporting periods.

ISH test-repeat rates for each laboratory are shown in Table 3. In the first 12 months the overall ISH test-repeat rate was 8.9 %, decreasing to 8.2 % in the second 12 months for laboratories using CISH. Twelve laboratories changed from using CISH to SISH in the second 12-month period. Repeat rates were higher (13.7 %) in these laboratories, although this was primarily caused by a global silver wash contamination issue that was subsequently addressed and resolved. In the third 12-month reporting period, test-repeat rates were 4.9 % for laboratories using CISH and 7.2 % for laboratories using SISH. In the final reporting period, test-repeat rates were 3.6 % for laboratories using CISH and 8.4 % for laboratories using SISH.

**Table 3:** Average ISH test-repeat rates recorded over time

Laboratory ID	Average ISH test-repeat rate, %			
	October 2006–September 2007	October 2007–September 2008	October 2008–September 2009	October 2009–September 2010
1^a^	6.6	22.2	20.2	13.7
2	–	–	1.8	5.2
3	9.0	6.6	6.3	0.0
4^a^	–	13.2	10.8	5.2
5^a^	6.7	3.7	3.5	8.4
6	2.0	1.6	0.7	0.8
7^a^	–	–	0.0	4.5
8^a^	–	10.5	1.4	3.4
9^a^	–	6.5	6.8	6.6
10^a^	8.4	18.7	22.7	26.8
11^a^	4.4	3.9	4.9	6.4
12^a^	16.8	14.7	6.1	13.5
13^a^	5.9	4.6	8.4	4.9
14^a^	8.7	8.8	3.1	9.4
15^a^	10.2	12.4	11.2	0.5
16	–	–	1.1	7.7
17	–	–	0.0	4.1
18^a^	5.5	4.2	2.9	7.8
19	23.5	14.3	5.0	2.1
20^a^	9.0	15.4	5.7	10.7
21	5.3	4.2	4.9	6.1
22^a^	15.9	13.7	5.9	2.1
23^a^	6.0	11.2	10.0	5.7
24	5.8	7.3	2.6	2.8
25^a^	10.6	11.5	4.1	0.4
26^a^	–	37.0	9.5	21.1

*ISH* in situ hybridization

^a^Laboratories using silver ISH for the final reporting period

### Retesting of discordant IHC/ISH cases

Of the 200 discordant cases selected, four were considered unsuitable for assessment due to the presence of considerable artifact(s), insufficient tissue, or loss or damage to the section during processing. Of the remaining 196 cases retested by IHC, 184 (94 %) showed concordance between the results of the repeat IHC and ISH. Eleven of the 12 cases that remained discordant (91.6 %) were false-negative and one was false-positive. The details of these 12 cases are shown in Table 4.

**Table 4:** Results of the 12 immunohistochemistry/in situ hybridization discordant cases

Case number	Local IHC	Central IHC	*HER2* gene copy no.^a^	*HER2*:CEP17 ratio	Comments
1	1+	1+	5.8 (f)	4.46	Low amp^b^
2	1+	1+	7.75 (f)	2.87	Low amp
3	1+	1+	8.15 (s)	Not done	Low amp
4	0	1+	7.35 and 1.25 (f)	2.94 and 1.9	Low amp
					Clonal
					Small clone of cells
5	1+	0	6.15 (f)	2.3	Low amp
6	1+	1+	7.75 (f)	4.31	Low amp
7	1+	1+	4.55 (f)	2.52	Low amp^b^
8	1+	1+	2.92 (f)	2.7	Low amp^b^ (Chr 17 monosomy)
9	1+	1+	3.95 (f)	2.47	Low amp^b^ (Chr 17 monosomy)
10	1+	1+	5.75 (f)	5.48	Low amp^b^ (Chr 17 monosomy)
11	1+	0	4.9 (f)	2.97	Low amp^b^
12	3+	3+	1.7 (f)	0.92	Non amp

*amp* Amplification, *CEP17* chromosome enumeration probe 17, *Chr* chromosome, *(f)* fluorescence in situ hybridization, *IHC* immunohistochemistry, *(s)* silver in situ hybridization (single probe for *HER2*)

^a^A negative result was defined by the investigators as <4 *HER2* gene copies, equivocal as ≥4 to <6 copies, low amplification as ≥6 to <10 copies, and high amplification as ≥10 copies

^b^Assessed as low amplification by the investigator due to a negative/equivocal result by *HER2* gene copy number and positive result by *HER2*:CEP17 ratio

An analysis of the cases that were now concordant following retesting showed that there were 45 cases reclassified from IHC 3+ to IHC 2+ (equivocal). Of those, 14 (31 %) had a chromosome 17 polysomy. Of the 161 cases that were originally scored as IHC 3+ but did not show gene amplification by ISH, 116 (72 %) were scored as IHC 0 or IHC 1+ after retesting.

## Discussion

Since the inception of the Australian In Situ Hybridization Program in October 2006, the number of HER2 ISH tests has increased each year; reflecting a shift toward HER2 ISH testing of all breast cancer samples (rather than the previous practice of triaging samples for ISH testing on the basis of IHC results). There was a 112 % increase in ISH testing reported between the first and last time period for patients with EBC, which is attributable to a greater understanding of the ISH testing program (i.e., all patients should be tested for ISH, regardless of IHC results), laboratory implementation of the ISH testing algorithm, and an increased number of laboratories qualified to report ISH. In addition, there is greater awareness among oncologists and breast surgeons that trastuzumab therapy should be available to all EBC patients with a positive ISH result. By comparison, ISH reporting of MBC cases was low and increased by just 20 % between the first and last reporting periods. Initial ISH testing of MBC cases is not a requirement of the Herceptin program administered by Medicare Australia. The smaller increase observed, may also reflect the fact that many patients presenting with MBC could have previously had their primary tumor tested for HER2 and therefore, may have received trastuzumab in the adjuvant setting [23]. In patients with EBC there was a reduction in HER2 positivity rates reported between the time periods (from 23.8 to 14.6 %) which reflects a shift toward the use of ISH testing for all samples without prior IHC triaging. The HER2 positivity rate of 14.6 % is comparable to rates reported in the literature [2–6, 24]. Although the average HER2 positivity rate among patients with MBC was higher than for EBC for all time periods and showed variations across the reporting period (22.6, 25.1, 21.3, and 21.6 % for the first, second, third, and fourth 12-month periods, respectively), these rates were also similar to those reported in the literature [2–6, 25, 26], suggesting that MBC is associated with a higher HER2 positivity rate than EBC and reflecting a more aggressive tumor cohort.

The decision to make trastuzumab therapy available to patients with HER2-positive EBC following an ISH-positive test is supported by recent guidelines for HER2 testing [27], which favor ISH over IHC due to its greater test accuracy, objectivity, and reproducibility. However, it should be noted that the use of ISH testing alone is associated with some risks, including an increased likelihood of failing to detect heterogeneity, overscoring highly polysomic cases (when a single probe is used), and missing cases with low *HER2* amplification. IHC is, therefore, a valuable tool for the assessment of equivocal or difficult cases and remains an important quality assurance measure. As such, we feel that the use of ISH testing, together with additional IHC testing as required, ensures the provision of accurate testing by all local laboratories, with a central laboratory providing further evaluation by FISH as necessary.

The efficiency of all laboratories involved in this nationwide program was illustrated by the consistently short overall reporting time for ISH tests, with average reporting times reduced slightly in the second 12-month period, despite the inclusion of four new laboratories and the fact that 12 laboratories switched to SISH testing. Average reporting times remained consistent in the third and fourth 12-month periods (4.6 and 4.5 days, respectively). For those laboratories that continued to use CISH, the test-repeat rates also decreased over the reporting period, reflecting the improvements in testing proficiency as a result of increasing experience. In the second reporting period, test-repeat rates were higher than expected for laboratories using SISH (13.7 %); however, this was attributed to a contamination of the silver wash which was reported in a number of countries outside Australia, and the test-repeat rate fell during the final two reporting periods to 7.2 and 8.4 %, respectively.

This study has demonstrated that there are inherent inaccuracies in local laboratory staining and/or assessment of HER2 IHC, where ISH is considered the “gold standard” test. This is highlighted by the fact that 72% of the 161 cases originally scored as IHC 3+ by local laboratories but found to be non-amplified by ISH were subsequently scored as IHC 0 or IHC 1+ by a central laboratory. However, our study has shown that very good concordance (94 %) exists between IHC and ISH when both tests are performed and interpreted by experienced laboratories and pathologists. There were a range of factors contributing to the discordance in the remaining 12 cases, including monosomy of chromosome 17 (three cases), and clonal amplification of *HER2* (one case). All of these cases showed only a low level amplification of the *HER2* gene (*HER2*:CEP17 ratio range 2.3–5.48). There is some debate regarding the relative importance of *HER2*:CEP17 ratio versus *HER2* copy number in assessing ISH [28, 29] and there was discordance between the two methods in 6/12 of our cases, reflecting the lack of an IHC 3+ score.

Although IHC/ISH discordance has been demonstrated previously in the setting of some clinical trials involving centralized retesting by FISH [20], our study has focused specifically on discordant cases. It remains unclear whether the laboratory test procedure, the pathologist’s interpretation, or both, contribute to the observed discordances. Therefore, a valuable additional analysis will be to compare results from small- and large-testing volume laboratories; however, this was not possible with the existing data.

The emphasis on accurate HER2 testing has been highlighted by the ASCO/CAP expert panel [18]. As well as recommending an updated scoring system for HER2 assessment, multiple factors that can cause variation in HER2 testing accuracy were identified, including fixation methods and assay reagents used [18]. Several standard assays exist for HER2 testing, which could result in a high degree of testing inaccuracy. The Australian In Situ Hybridization Program, as well as adhering to the ASCO/CAP HER2 testing guidelines, uses standardized HER2 testing kits (CISH, SISH, or DuoCISH) to minimize interlaboratory variation. All pathologists participating in the Australian In Situ Hybridization Program are required to perform a minimum number of 50 ISH tests annually, and each laboratory must perform a minimum of 150 tests annually. This ensures that there is a sufficient level of experience in participating laboratories. Participation in appropriate quality assurance programs is also mandatory. Further efforts to ensure the implementation of a highly accurate and robust HER2 testing system as part of this nationwide program included the emphasis on testing the excised tumor wherever possible, as testing on core biopsies may be less reliable [30]. Our data indicate that core biopsies were used for HER2 testing in <10 % of cases in most laboratories.

In summary, these findings demonstrate the successful implementation of a regulated, nationwide testing program that continues to collect data on HER2 testing in patients with breast cancer. We feel that the implementation of a high standard of training, accreditation, and quality assurance, as well as a streamlined approach to testing and reporting, have been fundamental to the success of this program. This methodology could be used as a model for the establishment of HER2 testing in other countries or for the implementation of other new biomarker-testing initiatives.

## Abbreviations

amp: Amplification
ASCO: American Society of Clinical Oncology
CAP: College of American Pathologists
CEP17: Chromosome enumeration probe 17
Chr: Chromosome
CISH: Chromogenic in situ hybridization
EBC: Early breast cancer
FISH or (f): Fluorescence in situ hybridization
HER2: Human epidermal growth factor receptor 2
IHC: Immunohistochemistry
ISH: In situ hybridization
MBC: Metastatic breast cancer
SISH or (s): Silver in situ hybridization
